# Novel Pharmacological Approaches to the Treatment of Depression

**DOI:** 10.3390/life12020196

**Published:** 2022-01-28

**Authors:** Elias Elias, Ariel Y. Zhang, Melissa T. Manners

**Affiliations:** Department of Biological Sciences, University of the Sciences, 600 South 43rd Street, Philadelphia, PA 19104, USA; eelias@mail.usciences.edu (E.E.); azhang0109@mail.usciences.edu (A.Y.Z.)

**Keywords:** antidepressant, natural products, major depressive disorder, pharmacological treatment, mental health, monoamine deficiency, opioid receptors, NMDAR, PPAR, mGluR, inflammation, HPA axis, microbiome, fatty acids

## Abstract

Major depressive disorder is one of the most prevalent mental health disorders. Monoamine-based antidepressants were the first drugs developed to treat major depressive disorder. More recently, ketamine and other analogues were introduced as fast-acting antidepressants. Unfortunately, currently available therapeutics are inadequate; lack of efficacy, adverse effects, and risks leave patients with limited treatment options. Efforts are now focused on understanding the etiology of depression and identifying novel targets for pharmacological treatment. In this review, we discuss promising novel pharmacological targets for the treatment of major depressive disorder. Targeting receptors including N-methyl-D-aspartate receptors, peroxisome proliferator-activated receptors, G-protein-coupled receptor 39, metabotropic glutamate receptors, galanin and opioid receptors has potential antidepressant effects. Compounds targeting biological processes: inflammation, the hypothalamic-pituitary-adrenal axis, the cholesterol biosynthesis pathway, and gut microbiota have also shown therapeutic potential. Additionally, natural products including plants, herbs, and fatty acids improved depressive symptoms and behaviors. In this review, a brief history of clinically available antidepressants will be provided, with a primary focus on novel pharmaceutical approaches with promising antidepressant effects in preclinical and clinical studies.

## 1. Introduction

Major depressive disorder (MDD) is one of the leading mental health conditions in the world. According to the World Health Organization, more than 264 million people are diagnosed with MDD worldwide [1]. MDD is a heterogeneous disease and is defined as a 2-week period during which a person experiences any combination of daily depressive symptoms including altered occupational and social functioning, altered mood, impaired daily activities and habits, loss of energy and interest in pleasurable tasks, weight changes, or sleep disturbances [2]. Most patients diagnosed with MDD report recurring depressive episodes [3], inducing suffering and increasing the risk of suicidality and death [4].

The development of the first antidepressant drugs in the 1960s was fueled by the monoamine deficiency hypothesis that monoamine neurotransmitter deficiency is the leading cause of MDD. This led to the development of the first generation of antidepressant monoamine oxidase inhibitors (MAOIs) and then tricyclic antidepressant agents (TCAs). Soon after, the role of serotonin in MDD was identified [5] and led to the development of current first-line therapeutics, selective serotonin reuptake inhibitors (SSRIs), and serotonin-norepinephrine reuptake inhibitors (SNRIs). There are notable shortcomings with classic antidepressants, including adverse events and delayed onset of efficacy [6]. Notably, over a third of patients do not respond to classic antidepressant treatment, which classifies these patients with treatment-resistant depression (TRD) [7]. More recently, the discovery of ketamine as an efficacious antidepressant led to the approval of a nasal spray form of esketamine [8]. Esketamine provides fast-acting symptomatic relief in TRD patients, yet the significant risks associated with esketamine limit its use for a broad patient population. Together, the adverse events, low efficacy, and dosing risks of currently approved medications leave MDD patients with inadequate treatment options. There are considerable challenges in treating patients with MDD due to the unique combination of symptoms each patient experiences. The etiological complexity of MDD indicates that this disorder is not a unitary disease. There are distinct pathophysiological mechanisms underlying susceptibility and resilience to the development of depression [9,10]. The majority of current antidepressants target monoamines, yet the limited responsiveness of MDD patients to these pharmacological treatments implicates that additional underlying mechanisms need to be considered in order to improve the efficacy of treatments [11]. Thus, expanding the currently available treatment options will work toward the goal of personalizing the pharmacological treatment plan of individual patients based on the specific considerations of their diagnosis.

In this review, we compiled the most up-to-date research on natural products, biological processes, and receptors that could serve as important novel targets to improve the outcomes of MDD treatment. Avenues to improve treatment options for patients include exploring novel targets, determining additional mechanisms of action of current antidepressants, or evaluating synergistic compounds for treatment. Therefore, the aim of this review is to provide a brief overview of currently available antidepressants and a detailed review of new molecular targets with promising potential for the treatment of MDD. In doing so, we suggest novel approaches that should be further explored for personalized antidepressant development.

## 2. Materials and Methods

The systemic literature search for this review was conducted in PubMed. The search was limited to publications written in English and publications assigned a PMID. Articles were chosen based on their relevance to the three major sections of this review, pharmacological agents targeting receptors or biological processes, or natural products with antidepressant potential.

## 3. Clinically Available Antidepressants

In this section, antidepressants that are currently available to patients are reviewed chronologically from when they were developed. Although these antidepressants are critical tools for the treatment of MDD patients, there are numerous limitations to their use: adverse effects, administration risks, delayed onset of antidepressant therapy, or limited efficacy. Therefore, in this section, we briefly review the efficacy of these drugs, potential off-label uses, and limitations for the treatment of MDD.

### 3.1. Monoamine Oxidase Inhibitors

MAOIs increase serotonin (5-HT or 5-hydroxytryptamine), norepinephrine and dopamine concentration in the synapse by inhibiting breakdown by monoamine oxidase A (MAO-A) and B (MAO-B). Iproniazid was the first MAOI used for the treatment of depression [12]. It was originally designed to treat tuberculosis; however, improved mood, stimulation of the central nervous system (CNS) and increase in 5-HT were also observed [13]. Iproniazid showed a 25% to 75% recovery rate in MDD patients and alleviated symptoms of atypical depression [14]. However, non-selective and irreversible binding resulted in a high concentration of tyramine, hypotension, and liver damage [15]. To improve MAOIs safety and efficacy, selective and reversible inhibitors of monoamine oxidase A (RIMA) were introduced in the 1980s. Moclobemide is a RIMA that has been widely used in MDD treatment [16].

Phenelzine, tranylcypromine, and isocarboxazid are also first generation MAOIs that are still used for TRD [17] in combination with other antidepressants [18,19]. Patients taking these drugs are required to limit tyramine intake to reduce the risk of developing hypertension [20].

Recent studies have focused on the off-label use of MAOIs for the treatment of Parkinson’s disease. These studies revealed that in animal models, selegiline and rasagiline improved synaptic plasticity by restoring long-term potentiation [21], and decreased cortical dopamine turnover time [22]. Improved motor function was observed in patients, indicating a promising therapeutic effect [23].

### 3.2. Tricyclic Antidepressants

Similar to MAOIs, TCAs also target the breakdown of monoamine neurotransmitters [17]. To enhance 5-HT and norepinephrine transporter (NET) function, TCAs desensitize 5-HT and NET presynaptic receptors by inhibiting the reuptake transporters and causing the accumulation of 5-HT and NET in the presynaptic cleft. In the absence of transport protein and postsynaptic breakdown, 5-HT and NET concentrations in the synaptic cleft increase, which is the presumed mechanism for improving mood to alleviate MDD symptoms [24]. Additionally, TCAs act as competitive antagonists on postsynaptic histamine, adrenergic and muscarinic receptors [17,18], which leads to various side effects of TCAs [25].

Imipramine, clomipramine (CLO), and doxepin (DXP) were among the first generation of TCAs produced [19,26]. Although these drugs have a common mechanism of action, there are advantages to each depending on the patient’s symptoms. CLO has a higher efficacy in treating MDD compared to imipramine [27]. DXP improves sleep in MDD patients [28,29] and is approved by the Food and Drug Administration (FDA) for insomnia treatment [30]. Nortriptyline is often prescribed to MDD patients with ischemic heart disease [31] as it reduces platelet activation [32].

Unfortunately, TCAs can have serious adverse effects. Antagonism of the postsynaptic histamine, adrenergic and muscarinic receptors, caused different levels of adverse effects such as drowsiness [33], pregnancy complications [34], and cardiac abnormalities such as abnormal cardiac conduction, arrhythmia, and myocardial infarction [35,36]. In utero exposure to CLO caused withdrawal symptoms including convulsion, increased heart rate, and cyanosis within 24 h post-partum [37]. More efficacious treatments such as SSRIs or SNRIs are available and therefore, TCAs are no longer the first choice for antidepressant treatment [38].

Research on the use of TCAs for chronic migraine [39], insomnia [40,41], alcohol use disorder [42,43], and neuropathic corneal pain has been conducted with indications of therapeutic efficacy, though adverse effects remain a concern.

### 3.3. Selective Serotonin Reuptake Inhibitors

In the 1960s, researchers uncovered the potential role of serotonin in MDD [5]. One of the suggested therapeutic approaches was the inhibition of serotonin reuptake to enhance stimulation of postsynaptic serotonin receptors, which led to the development of SSRIs. Fluoxetine was the first SSRI to be FDA approved, followed by numerous SSRIs with reduced adverse effects. Although SSRIs produce an increase in serotonin in the synapse, there is a delayed onset of efficacy, which can be 2–3 weeks in duration [6]. Investigation into the mechanism of antidepressant action expanded from increasing serotonin in the synapse to increasing hippocampal neurogenesis [44,45,46].

Because of the high tolerance and broad-spectrum efficacy, SSRIs are prescribed as a first-line medication for depressed patients of different ages. However, patients reported adverse events including headache, agitation, nausea, diarrhea, insomnia [47], sexual dysfunction [48], gynecomastia [49], weight gain, and gastrointestinal distress [50].

The primary goal of current research is to identify key factors needed to minimize adverse effects and delayed onset of therapeutic action. SSRI-induced upregulation of the brain-derived neurotrophic factor (BDNF) [51] and dopamine receptor D1 [52], reduction of inflammatory factors involved in the c-jun N-terminal kinase 1/2 and the extracellular signal-regulated protein kinases 1 and 2 (ERK1/2) pathways [53,54], or phosphorylation of the mammalian target of rapamycin (mTOR) [55] could be involved in the antidepressant action of SSRI. The unique molecular mechanisms underlying the activity of SSRIs may be an important consideration for determining the cause of adverse effects and delayed onset of efficacy, and could lead to the development of improved therapeutics.

### 3.4. Serotonin-Norepinephrine Reuptake Inhibitors

SNRIs inhibit the reuptake of two important neurotransmitters involved in depression: serotonin and norepinephrine. The first developed SNRI, venlafaxine, was FDA approved and marketed in the United States of America (USA) in 1993 as a first-line treatment for depression [56]. Over the following decade, desvenlafaxine [57], duloxetine [58], levomilnacipran [59], milnacipran [60], and other SNRIs were also approved. Treatment of depressed adult patients with levomilnacipran increased thickness in the left insular cortex [61]. This link between levomilnacipran and cortical thickness has been studied in previous clinical trials, but the underlying mechanism of action has not been elucidated.

Duloxetine is an SNRI with the potential to treat neuropathic pain. Duloxetine decreased chemotherapy-induced nerve pain by inhibiting p53 [62], p38 phosphorylation, and NF-κB activation [63]. Because chronic pain and depressive disorders are frequent comorbidities, clinical trials should explore the potential of SNRIs to treat neuropathic pain conditions while preventing depressive symptoms in these patients.

Side effects of SNRIs are similar to SSRIs, including tiredness, constipation, insomnia [64], and potentially the dysregulation of some metabolic processes associated with hyperglycemia [65,66]. Despite the observed success in treating MDD patients, first-line therapeutics are not effective in many patients, resulting in multiple adverse effects and delayed efficacy [67]. This shifted the therapeutic need to drugs that are fast-acting, with reduced adverse effects and improved efficacy for TRD.

### 3.5. Ketamine

Ketamine, a phencyclidine derivative, is a noncompetitive N-methyl-D-aspartate receptor (NMDAR) antagonist developed as an anesthetic in the 1960s. NMDARs are heteromeric ionotropic glutamatergic receptors that are activated by the binding of both glycine and glutamate to their corresponding binding site (NR1 and NR2 subunits respectively) and are inhibited by magnesium ions binding to the phencyclidine site. Thus, ketamine antagonistic activity is delivered by binding to the phencyclidine site or by non-selectively binding to the NR2 site (A-D subunits) [68,69,70].

The first clinical trial that tested the therapeutic potential of ketamine as an antidepressant was conducted in 2000 [71]. Delivering its effects in as little as 30 min for patients with TRD [72,73,74] or bipolar depression [75], ketamine was considered the first fast-acting antidepressant. Administration of ketamine to patients is problematic and impractical; antidepressant effects are short acting, and the lack of protracted effects require frequent intravenous administration by an anesthesiologist [3].

There are two active enantiomers of ketamine, both with antidepressant activity, R-ketamine (also known as arketamine) and S-ketamine (also known as esketamine). Despite the lower affinity of arketamine to NMDAR, preclinical models identified enhanced protracted antidepressant effects [76,77,78], which were confirmed in a recent clinical trial [79].

In 2019, a nasal spray form of esketamine was approved by the FDA in the USA and Europe [80]. MDD and TRD patients who received esketamine alone or in combination with standard antidepressants had significant improvement in MDD symptoms compared to standard antidepressants [80], with high tolerability and safety [81]. Additionally, this enantiomer is used for patients with severe illness or active suicidal thoughts [82].

There are continuing safety concerns that restrict the usage of esketamine due to multiple adverse events including hyperhidrosis, dissociation, urinary pain, and suicidal ideation [83,84,85]. Therefore, extensive research is being conducted to study key factors involved in the mechanism of action, with the goal of developing new and improved therapeutics for MDD treatment that are fast-acting and long-lasting, with reduced adverse effects and abuse liability. Some proposed mechanisms include regulation of the neuropeptide precursor VGF [86], stimulation of serotonin1A receptor [87], activation of the BDNF pathway, induction of the α-amino-3-hydroxy-5-methyl-4-isoxazolepropionic acid receptor (AMPAR) [88], and activation of the ventral CA3 hippocampal regions [89].

### 3.6. γ-Aminobutyric Acid

γ-Aminobutyric acid (GABA) is a neurotransmitter that activates two classes of receptors: the ionotropic and metabotropic receptors (GABA_A_Rs and GABA_B_Rs), mediating neural inhibition in the CNS [90,91]. Ionotropic receptors are targeted by allosteric modulators benzodiazepines (Bz) and barbiturates [92] to produce an anxiolytic effect. Metabotropic receptors regulate excitatory postsynaptic potentials and have been considered in affective disorders.

The GABAergic system is dysregulated in depressive patients. GABA synthesizing neurons are decreased in the orbitofrontal cortex of MDD patients [93], cortical GABA is downregulated in postpartum depression [94], and TRD patients have decreased binding of Bz in the frontal and orbitotemporal cortex [95]. Following SSRI treatment, depressive patients have increased cortical GABA [96], and upregulated levels of allopregnanolone and pregnanolone, two GABA-active steroid agonists [97].

GABAergic drugs that are predicted to have antidepressant activity from preclinical studies included valproate [98], amino-oxyacetic acid [99], beta-phenyl-GABA [100], muscimol [101], diazepam [102], carbamazepine, and oxcarbazepine [103].

More evidence for the role of the GABAergic system in depressive disorders is its involvement in the serotoninergic system [104] and its contribution to the ketamine response through glutamate synaptic changes or cortical excitatory-inhibitory balance reestablishment [105,106]. GABA_B_ receptors are important for the antidepressant activity of ketamine [107], and other GABA_B_ agonists such as baclofen, CGP7930, and SKF97541 showed significant antidepressant activity [108].

One of the remaining challenges is investigating the extent to which the GABAergic system dysregulation or imbalance impacts specific subsets of MDD patients. Given the widespread coverage of GABAergic neurons, their innervation in other modulatory systems, and the strong link between modulating GABA and the efficacy of antidepressants, the GABAergic system remains a critical area of research and could be important in achieving the antidepressant activity of potential future therapeutics.

## 4. Potential Antidepressants Targeting Receptors

One approach to developing new treatments for MDD is targeting specific receptors and receptor families that are implicated in the pathophysiology of depression. In this section, we review preclinical and clinical reports that target specific classes of receptors for the treatment of depression. Receptor families discussed below have been understudied in the field of depression and demonstrated promising novel treatment options. This section, Supplementary Tables S1 and S2 summarize agonists, antagonists, and other regulators that could lead to promising potential antidepressants.

### 4.1. Opioid Receptors

The delta (DOR), kappa (KOR), and mu (MOR) opioid receptors are endogenous G protein-coupled receptors. They are expressed throughout the nervous system and regulate important physiological functions such as stress response, reward process, and mood. Opioid receptors are activated via binding of endorphin, enkephalin (ENK), dynorphin, and nociceptin/orphanin FQ, and have numerous downstream effects. Modulating the opioid receptors has antidepressant potential, through specific or non-specific agonists and antagonists.

Buprenorphine (BUP) is a partial MOR agonist and a DOR and KOR antagonist, with a high affinity for MOR and KOR and a lower affinity for DOR [109,110]. It was originally approved as an analgesic to treat moderate to severe pain [111] and withdrawal symptoms from opioid dependence [112]. For the last several decades, it has been administered when patients did not respond to conventional antidepressants [113]. It improved mood, reduced anxiety behavior, and suicidal ideations [114]. It also rapidly improved TRD symptoms within the first three weeks with no serious side effects [115]. Combinatorial treatment of BUP/samidorphan was also efficacious and was well tolerated in depressed patients with no signs of abuse or opioid dependence/withdrawal [116,117]. Similar to BUP, nalmefene targets multiple opioid receptors and was shown to prevent inflammation-induced depressive-like behaviors in rodents and cognitive impairment by decreasing hyperactivity in the hypothalamus-pituitary-adrenal (HPA) axis and increasing BDNF expression [118]. More studies should be conducted to determine if nalmefene has therapeutic potential in humans.

There is also an opportunity to develop therapeutics targeting specific opioid receptors for the treatment of MDD. ENK is a pentapeptide that binds to DOR and MOR, and has been used as an analgesic since the 1970s [119]. Chronic mild stress upregulated ENK production and activated the MOR and DOR signaling pathway, while ENK knockout mice did not express depressive-like or anxiety-like behaviors. This finding suggests that inhibition of ENK promotes stress resilience and that this pathway is a potential therapeutic target for MDD [120]. KNT-127 [121], Rubiscolin-6 [122], and SNC80 [123] are preclinical DOR receptor agonists that decreased depressive-like behaviors in mouse models of depression. N/OFQ is an endogenous opioid peptide that binds to nociceptin receptor (NOPr), part of the opioid receptor family [124]. NOPr antagonists such as LY2940094 [125] and UFP-101 [126] reduced anhedonia and rescued stress-like and anxiety-like behaviors by restoring neurogenesis after chronic stress.

Tianeptine is classified as a μ-opioid receptor agonist that has been shown to have antidepressant effects, with no negative effects on sleep, cognitive function, or memory [127]. In rodents, tianeptine reduced depressive-like behaviors and inflammatory factors in the hippocampus and prefrontal cortex (PFC) [128], reduced avoidant behavior [129], and had positive effects on energy and metabolic processes [130]. A number of studies have reported the enhanced efficacy of tianeptine when combined with another pharmacotherapy. Mice administered tianeptine and cannabinoid targeting drugs had reduced depressive-like behaviors [131]. Ramoz et al. recently reported an association between the antidepressant effects of tianeptine and the corticotropin-releasing hormone receptor in patients experiencing major depressive episodes [132].

It is unknown if tianeptine provides improved antidepressant effects compared to first-line antidepressant treatments. However, the combined clinical and preclinical evidence suggests that further clinical studies should be conducted to ascertain whether tianeptine should be considered for individual or combinatorial treatment for MDD patients.

Opioid receptor-targeting treatments have been effective for patients with TRD [133], which indicates that TRD patients require molecular targets unique from monoamines. Given the involvement of opioid receptors in stress, reward, mood, and biological and cellular processes such as inflammation, it is expected that targeting this family of receptors would be efficacious for treating psychiatric diseases [134,135,136]. However, targeting the opioid system is problematic as chronic usage has an abuse potential, and the development of tolerance or withdrawal symptoms may occur. Therefore, the major challenges in developing opioid targeting drugs have likely hindered progress in the development of novel therapeutics.

### 4.2. N-methyl-D-aspartate Receptors

Increased glutamate is also associated with MDD. The binding of glutamate activates NMDAR and mediates cell depolarization. AMPAR is another type of ionotropic and glutamatergic receptor. The density and stability of AMPAR are crucial for synaptic transmission [137] and can mediate antidepressant effects upon activation [138]. Both preclinical and clinical studies have extensively focused on NMDAR antagonists for the treatment of depression. In this section, we focus on non-ketamine related potential therapeutic targets for the treatment of MDD.

Nitrous oxide (N_2_O) is an NMDAR antagonist that has antidepressant properties similar to ketamine. N_2_O activated neuronal nitric oxide synthase and restored synaptic plasticity [139]. N_2_O exposure reduced depressive-like behaviors, increased BDNF expression, and neuron firing rate in the medial PFC of mice [140]. N_2_O also demonstrated a rapid antidepressant effect in TRD patients with a high treatment response rate [141] and significantly improved Hamilton Depression Rating Scale scores in MDD patients with a high remission rate. Although N_2_O may be promising for TRD treatment, there are obstacles for utilizing this treatment such as side effects [142] and inconvenient administration.

Memantine, another NMDAR antagonist, was effective in treating MDD patients over 60 years old in combination with standard SSRI treatment. Importantly, no major side effects were reported [143], indicating that memantine is a potential therapeutic agent for combinatorial therapy with SSRI in aging adults with MDD. Further investigation to determine if effects are age-specific is needed to evaluate the potential limitations of this treatment. Riluzole inhibited post-synaptic glutamic release by noncompetitive blockade of NMDAR and exhibited a rapid antidepressant effect in patients with moderate to severe MDD [144].

A number of other NMDAR antagonists has promising behavioral effects in rodents. Amantadine, which is used as an antiviral, is an NMDAR antagonist that was shown to have antidepressant and anti-inflammatory effects [145]. Lanicemine and MK-801 are NMDAR antagonists that rapidly rescued stress-induced depressive-like behaviors [146,147,148]. Ro 25-6981, a selective NMDAR inhibitor with high oral permeability, reduced depressive-like symptoms in rodents, indicating the potential to develop orally administered NMDAR antagonists [149].

The GluN2B subunit is one of the major components in NMDAR and plays functional roles in NMDA signaling [150]. Blockage of GluN2B and inhibition of GluN2B phosphorylation exerted rapid antidepressant-like effects in animal models [151,152]. Pharmacological studies are being conducted to determine the mechanism of action of the GluN2B subunit. Ifenprodil, a GluN2B selective antagonist, reduced depressive-like behaviors through mTOR activation [153]. Rislenemdaz decreased GluN2B activity and BDNF expression in the lateral habenula [154]. An NMDAR positive allosteric modulator, AGN-241751, rapidly promoted antidepressant behavior by enhancing GluN2B-NMDA signaling and increasing the concentration of synaptic protein in the medial PFC [155]. Traxoprodil, a GluN2B antagonist, reduced depressive-like behaviors and reinforced the antidepressant effect elicited by conventional MDD treatments such as desipramine, paroxetine, milnacipran, and bupropion [156,157].

NMDAR antagonists have delivered rapid antidepressant effects in pre-clinical models and clinical evidence confirms these effects, though more clinical trials are necessary to verify efficacy. Specifically targeting the GluN2B subunit of NMDAR is a novel approach for delivering fast-acting symptom reduction and could be utilized as an alternative to ketamine-derived drugs for patients with suicidal ideation. Further exploration on combinatorial therapy of NMDAR antagonists with SSRI or SNRI could provide fast-acting symptom relief from the NMDAR antagonist and sustained antidepressant activity from SSRI or SNRI. Other NMDAR antagonists such as memantine and amantadine require rigorous clinical trials to determine if they can be used to treat TRD.

### 4.3. Peroxisome Proliferator-Activated Receptors

The peroxisome proliferator-activated receptors (PPARs) are transcription factors that play a role in lipid metabolism, glucose regulation, energy homeostasis, and inflammation. Decreased levels of the PPAR subtypes (α, β/δ, and γ) have been associated with depressive behaviors, and targeting these receptors is of therapeutic interest [158].

Knockdown of hippocampal PPAR-γ was linked with behavioral changes, reduced neurogenesis, and neuronal differentiation in mouse models [159]. Disrupting the regulation of PPAR-δ by non-coding RNA promoted depressive-like behaviors [160]. Interestingly, venlafaxine, an SNRI, has been shown to require activation of PPAR for antidepressant effects [161]. Because the reduced activity of PPAR is associated with depressive behaviors, increased expression and activation of PPAR have been the focus for antidepressant treatment.

PPAR-γ agonists used in combination with simvastatin improved behavioral effects and had anti-inflammatory effects in rodent models of depression. Pioglitazone is a PPAR-γ agonist that normalized the stress-induced increase in tumor necrosis factor alpha (TNF-α), interleukin 6 (IL-6), and interleukin 1-beta (IL-1β) expression levels, and in hippocampal astrocytic activation [162]. It also decreased depressive-like behaviors in a lipopolysaccharide (LPS) model [163]. Rosiglitazone is another PPAR-γ agonist that attenuated neuroinflammation in stressed mice [164], suggesting that both agonists had antidepressant and anti-inflammatory activity. Interestingly, expression data from MDD patients showed that PPAR inhibits inflammatory factors and reactive oxygen species [165].

PPAR is involved in neurogenesis and cellular and behavior function that is associated with depression, therefore modulating PPAR could be an important therapeutic avenue for further research [166]. Using PPAR agonists with either statins or SNRIs improved the anti-depressive activity of these drugs in preclinical models, which proved to be an important mechanism in their antidepressant activity [161,162,163]. The development of PPAR agonists should be strongly considered. Combinatorial therapy should be further explored in the clinic, where individuals who are already administered SSRI, SNRI, or statins may benefit from adding a PPAR agonist to their regimen.

### 4.4. G-Protein-Coupled Receptor 39

Zinc deficiency and decreased expression of the zinc sensing receptor G-protein-coupled receptor 39 (GPR39) are associated with MDD [167]. When GPR39 was silenced in a mouse model, increased anxiety-like and depressive-like behaviors, increased passive coping, and treatment resistance to MAOI were observed [168]. On the other hand, there is evidence that agonizing the GPR39 receptor is associated with antidepressant effects. The GPR39 agonist, TC-G 1008, induced prolonged antidepressant-like effects compared to acute administration of imipramine, ZnCl2, or the NMDAR antagonist MK-801 [169]. Ghrelin is a hunger-associated hormone and endogenous GPR39 agonist. Ghrelin administration reduced neuroinflammation and alleviated depressive-like behaviors induced by myocardial infarction in rats [170]. These data indicate that supplementing zinc [171] or targeting the zinc sensing receptors are potential approaches to treat MDD. However, zinc supplementation has been accompanied by adverse events including chills, fever, vomiting, heartburn, and shortness of breath [172]. These symptoms are similar to those reported by clinically available antidepressants [47,64] and could result in enhanced adverse effects if combined with other antidepressants. More preclinical studies are necessary to confirm the mechanisms underlying behavioral findings.

### 4.5. Metabotropic Glutamate Receptors

Metabotropic glutamate receptors (mGluRs) are G protein-coupled receptors that mediate glutamate response through second messenger systems. Targeting specific subtypes of mGluRs has shown antidepressant potential.

mGluR5 localizes in the postsynaptic membrane and modulates synaptic excitability and plasticity. mGluR5 played a crucial role in long-term depression (LTD) by mediating paired-pulse low-frequency stimulation LTD in the hippocampus [173]. It increased excitability and NMDAR activity that is often associated with anxiety and hyperactivity [174], and mediated the rapid antidepressant effect of ketamine [175]. mGluR1/5 might play an important role in regulating synaptic activity. Depressive-like behaviors were observed in mice when the retinoid X receptor gamma, the receptor that promotes mGluR1/5 mediated response, was silenced in mice [176]. In a mGluR5 knockout model, mice lacking mGluR5 were more susceptible to depressive-like behavior. Rescuing mGluR5 expression in the nucleus accumbens decreased depressive-like behaviors in mice [177]. This finding emphasizes the crucial role of mGluR5 expression in stress resilience and the potential of targeting mGluR5 as a mechanism of antidepressant action. Interestingly, in a clinical trial, basimglurant, a selective negative mGluR5 allosteric modulator improved Montgomery-Asberg Depression Rating Scale, and Depressive Symptomatology–Self-Report of MDD patients at the secondary endpoint of the trial [178].

mGluR2/3 is elevated in animal models of chronic stress with neuronal damage in the hippocampus, suggesting that the mGluR2/3 pathway promoted neuronal damage and neuron viability [179]. Reducing the activity of mGluR2/3 using negative allosteric modulators [180] or mGluR2/3 antagonists, LY341495 [181] and LY3020371 [182] produced antidepressant effects in mice. Interestingly, the combination of (2R,6R)-HNK, a ketamine metabolite, with LY341495 elicited the same antidepressant effect as ketamine with reduced adverse effects [183].

mGluRs-mediated modulation on synaptic transmission can potentially have a protective effect on susceptibility to the effects of chronic stress [174]. mGluRs can also be used as an adjuvant or in combinatorial therapy to reduce the side effects of ketamine-based antidepressant therapy without hindering the efficacy of ketamine [183]. These studies suggest that mGluRs may have both a protective and therapeutic effect against the development of depressive behaviors. Clinical trials are necessary to determine the efficacy of mGluR treatments alone or with other clinically available antidepressants.

### 4.6. Galanin Receptors

Galanin is a neuropeptide commonly found in neurons of the CNS that binds to three different galanin receptor (GALR) subtypes that can form hetero or homodimers. GALR2 mediates excitatory signaling while GALR1 and GALR3 inhibit it. Activation of GALR2 has antidepressant effects, while GALR1 and GALR3 enhanced depressive-like behaviors [184,185,186].

Recently, investigation on the underlying molecular mechanism of these receptors in depression has involved using the synthesized galanin analog GAL(1–15), an active N-terminal fragment of galanin [187]. GAL(1–15) promoted depressive-like behaviors in mice by binding to the GALR1-GALR2 heterodimer [188]. However, administration of the 5-HT1AR agonist 8-OH-DPAT, or SSRI fluoxetine [189] in combination with GAL(1–15), reduced depressive-like behaviors, and this effect was blocked by inhibition of the 5HT1AR [190]. The GALR2 antagonist, M871, increased depressive behaviors and increased anxiety-like behaviors in rats [191] while the administration of the GAL2 agonist AR-M1896 [192] or an active galanin analog [193] produced antidepressant effects. Altogether, this indicates that targeting GALR1-GALR2-5-HT1AR heteroreceptor complexes might be a promising future therapeutic strategy to treat depression.

## 5. Potential Antidepressants Targeting Biological Processes

Another approach to the development of antidepressant treatment is to target broad pathways and biological processes instead of specific receptors. In doing so, dysregulated cellular and physiological effects can be treated. In this section, we focus on biological processes that are known to be involved in the development of depression, such as inflammation, the HPA axis, cholesterol biosynthesis, and gut microbiota. We focus on reports that indicate strong antidepressant potential. These agents and processes are discussed in this section and summarized in Supplementary Tables S3 and S4.

### 5.1. Inflammation

There is a strong link between inflammation and depressive disorders. MDD patients often have an elevated immune response [194] and pro-inflammatory factors [195] such as TNF-α [196], C-reactive protein (CRP), IL-6, and interleukin-1 receptor antagonist (IL-1ra) [197], while patients with somatic and inflammatory disorders have an increased risk of developing MDD [198]. Patients who received pro-inflammatory treatments for somatic diseases are likely to develop depressive symptoms [199]. This bidirectional relationship between inflammation and MDD indicates that anti-inflammatory agents are a potential therapeutic for MDD.

Currently used anti-inflammatory drugs have produced antidepressant effects in patients. Clinical trials on the cyclooxygenase (COX)-2 inhibitor indicate that anti-inflammatory agents can reduce depressive symptoms in MDD patients [200]. The release of cytokines promotes an increase in COX-1 and COX-2 activities, leads to prostaglandins synthesis, and further prolongs the inflammation [201]. Non-steroidal anti-inflammatory drugs (NSAIDs) inhibit COX-1 and 2 and prevent the cascade of COX enzymatic reactions [202].

Preclinical studies have suggested that inhibitors of COX-2 have antidepressant effects in animal models. Lumiracoxib is a COX-2 inhibitor that normalized corticosterone-induced glutamatergic currents in the amygdala, reducing anxiety-like behaviors in mice [203]. Nimesulide, a COX-2 inhibitor with antioxidant and anti-inflammatory properties induced antidepressant-like effects and downregulated the production of inflammatory factors induced by oxidative stress [204].

In clinical studies, both COX-1 and 2 antagonists have been effective in reducing depressive symptoms. Diclofenac improved depressive symptoms in chronic pain patients [205]. Celecoxib, a selective COX-2 inhibitor, combinatorial treatment with SSRI twice a day for 8 weeks was more effective than SSRI alone in alleviating depressive symptoms of MDD patients [206]. Improved cognitive function and emotion processing after six weeks of treatment have also been reported [207].

Over-the-counter NSAIDs including ibuprofen, aspirin, and naproxen have been associated with antidepressant effects. Ibuprofen is a COX-2 inhibitor that is commonly used to treat pain and fever [208]. Ibuprofen reduced inflammation [209], and chronic stress-induced depressive-like behaviors [210] in mice by downregulating BDNF in the hippocampus [211]. Aspirin is an NSAID that inhibits COX-1. Depressive patients treated with a combined treatment of aspirin and minocycline had a significant improvement in their clinical response rate, indicating that aspirin has the potential to enhance antidepressant efficacy [212]. Naproxen is another over-the-counter NSAID that reduces pain and fever by non-selective inhibition of both COX-1 and COX-2 [213] that reduced symptoms in osteoarthritis patients [214]. Interestingly, neither aspirin nor naproxen produced antidepressant effects in elderly patients [215,216], indicating that there the limitations to this treatment that should be further defined.

Clinical trials on the TNF-α inhibitor etanercept indicate that additional anti-inflammatory agents can also reduce depressive symptoms in MDD patients [217]. Infliximab, a TNF-α antagonist, inhibited TNF receptor (TNFR)/nuclear factor κB (NF-κB) signaling and depressive symptoms in MDD patients who were exposed to early life stress [218]. Minocycline is a tetracycline antibiotic that mediated neuroprotective effects and alleviated depressive symptoms by modulating CRP_and IL-6 [219,220,221]. Medications used to treat inflammatory conditions, such as psoriasis, (guselkumab [222] and ustekinumab [223]), rheumatoid arthritis (etanercept [224] and tocilizumab [225]), hidradenitis suppurativa (adalimumab [226]) also improved depressive symptoms in these patients.

Administration of N-acetyl cysteine (NAC), an antioxidant, to mice reduced depressive-like behaviors and restored the corticosterone levels in the amygdala [227]. It also provided a neuroprotective effect against oxidative stress and DNA damage in the hippocampus due to NAC antioxidant and anti-inflammatory effects [228]. Similarly, modafinil, an anti-epileptic medication, exerted neuroprotective effects through its anti-inflammatory properties [229].

The strong association of inflammatory diseases, inflammation, and depression emphasizes the importance of the inflammatory system in depression and the deep connection of systemic and neuroinflammation with mental health [230]. Utilizing the appropriate anti-inflammatory agents to manage chronic inflammatory diseases may be critical to prevent depression as a comorbidity. It may also be prudent to reduce systemic inflammation for acute inflammatory indications to prevent the development of depressive symptoms [231]. Utilizing anti-inflammatory agents to reduce heightened basal inflammatory levels should also be further explored in depression cases independent of inflammatory disease. Targeting specific inflammatory cytokines, the NF-κB signaling pathway, or targeting COX could be potential therapeutic strategies to treat a broad population of patients [218,232]. The unique reactivity of the inflammatory system could be one aspect of why some people are more prone to MDD than others.

### 5.2. Hypothalamic-Pituitary-Adrenal Axis

HPA axis is the major neuroendocrine system in the CNS that mediates the stress response [233]. Prolonged stress may lead to hyperactivation of the HPA axis and increased production of the corticotropin-releasing hormone in the positive feedback loop, suggesting that blocking the hyperactivation of the HPA axis is a key factor in preventing stress-induced depression [234,235].

The mineralocorticoid receptor (MR) and glucocorticoid receptor (GR) regulate the HPA axis by triggering the stress response. MRs are primarily expressed in the hippocampus and PFC and play a major role in memory and executive function [236]. Genetic variants of MR have been associated with susceptibility or resilience to depressive symptoms. Individuals with certain haplotypes are more susceptible to early life stressors [237] or have sex-dependent susceptibility or resilience to childhood trauma [238] and to the development of MDD [239].

There is an inverse correlation between MR and depression; chronic stress downregulates MR and antidepressants increase MR expression. Increased MR decreases the activity of the HPA axis, to promote reduced anxiety and stress-coping mechanisms [240]. Depressive patients treated with an MR agonist, fludrocortisone, had significant improvement in executive function and memory. Importantly, this mechanism resulted in stimulation of MR without impairing cortisol secretion from the adrenals, which could cause numerous and widespread adverse effects [241]. MR antagonist, spironolactone, decreased stress-induced amygdala activity and improved cognitive function in MDD patients [242], but TRD patients were unresponsive to treatment [243] indicating that MR dysfunction could contribute to TRD. However, over-activation of MR increases the risk of prolonged inflammation in the brain [239], which increases M1 microglia. Since chronic stress induces remodeling of M1 microglia in the PFC [244], this could contribute to the development of MDD and pathological damage in the brain [245]. To maintain homeostasis in the brain, GRs are expressed to dampen MR function by reducing inflammation. This reflects the importance of the MR:GR ratio in the brain, and the inherent risks with targeting MR to treat MDD [246].

Glucocorticoid (GC) is a hormone synthesized and regulated by the HPA axis in response to stress and mediated by GR [247]. Similar to MRs, impaired or dysregulated GRs can lead to the development of MDD [248]. The regulation of GRs by the Abelson helper integration site 1 (Ahi1) gene is involved in depressive behaviors. A heterozygous Ahi1 knockout mouse exhibited stress resilience [249], while a homozygous knockout of Ahi1 showed depressive-like symptoms [250], and reduced the efficacy of antidepressants [251]. FK506 binding protein 51 (FKBP5) is another GR regulator that prevents GC binding, leading to hyperactivation of the HPA axis [252]. FKBP5 knockout mice displayed HPA axis inhibition with depressive-like behaviors induced by chronic restraint stress [253]. High expression of FKBP5 was positively correlated with increased depressive symptoms and reduced antidepressant effects in patients. Overexpression of GRs has been associated with stress resilience in animal models. Rodents overexpressing MR were less likely to develop helplessness behaviors compared to rodents with reduced GR expression [254,255]. Clinically, GR agonists prednisone and dexamethasone lowered cortisol levels in MDD patients who experienced early life stress [256]. GR regulators should be further considered as potential novel therapeutics for the treatment of depression.

Increased activity of the HPA axis also leads to psychotic major depression (PMD) [257]. Compared to MDD patients, higher cortisol levels and hyperactivity of the HPA axis were reported in PMD patients [258]. Mifepristone, a GR antagonist, decreased psychotic symptoms and cortisol levels specifically in PMD patients [259]. Mifepristone could also treat alcohol dependence-induced depression and cognitive deficits with no major side effects [260].

Arginine vasopressin type 1B receptor (V1B) has also been a therapeutic target in MDD treatments. ABT-436, a V1B antagonist, decreased saliva cortisol, serum cortisol, and free cortisol levels of depressed patients 8 h after administration, and alleviated depressive symptoms [261]. Another V1B antagonist, TS-121, decreased cortisol levels and depressive symptoms in TRD patients with hyperactive HPA axis [262].

The HPA axis has a defined role in the stress response and in the development of depression. Inhibition of the HPA axis or modulation of MRs and GRs can play a role in resilience to chronic stress [254]. Preclinical models manipulating the expression of MR and GR show promising results, however, this is a difficult concept to mimic in the clinic. Therefore, future studies should use haplotype information to stratify patient populations for alternative treatment of depression or determine if preventative therapies are suitable for those susceptible to the development of MDD. Stimulation of GR could be a potential therapy to treat MDD patients specifically exposed to early life stress [256]. However, the safety and long-term efficacy of GR stimulation are unknown. Although suppression of MR reduced stress-related amygdala activities, it was not effective in TRD patients reflecting a limitation to this potential treatment [243]. Drugs targeting MRs and GRs have not been explored to their fullest potential. Therefore, one potential avenue is to determine the safety and efficacy of targeting these receptors.

### 5.3. Cholesterol Biosynthesis Pathway

Statins are hydroxymethylglutaryl-CoA reductase inhibitors. Since this enzyme plays an important role in the cholesterol biosynthesis pathway, statins possess a hypolipidemic effect and are thus prescribed to patients with cardiovascular disorders [263]. Statins were clinically shown to reduce inflammation with a fast and early efficacy [264]. Recently, numerous preclinical and clinical studies tested the potential antidepressant and neuroprotection effects of these inhibitors.

Simvastatin reduced depressive-like behaviors in mice and downregulated glial activation and pro-inflammatory cytokines [265]. This suggests that the antidepressant potential of simvastatin could be mediated by a decrease in neuroinflammation and by the inhibition of the NF-κB pathway [265]. This antidepressant effect was also observed in high-fat diet mice along with an attenuation of neuroinflammatory factors in the hippocampus [266]. In ovariectomized rats, simvastatin decreased depressive-like behaviors, downregulated the expression of toll-like receptors 2 and 4, reduced IL-1β and IL-18 expression, and limited microglia activation in the hippocampus [267]. When co-administered with an opioid receptor antagonist (naloxone), the antidepressant effect of simvastatin was reduced, suggesting that opioid receptors could be involved in statin’s mechanism of action [268]. Atorvastatin decreased depressive-like behaviors in several mouse models of depression by decreasing TNF- α, IL-1β, and NF-κB p65, or by increasing hippocampal BDNF [269,270,271].

The antidepressant effect of atorvastatin and simvastatin are dependent on the agmatine and imidazoline receptors; pretreatment of mice with an agmatine receptor agonist potentiated the effect of both statins, while the blockade of imidazoline receptors reduced efficacy [272]. Similar to atorvastatin, lovastatin might be preferred for patients with diabetes and depressive symptoms [273].

Statins are one of the most widely prescribed drugs in the world [274], yet we do not fully understand their potential in reducing depressive symptoms. With a large number of patients taking statins throughout the world, future studies should collect data to determine if there are mood-altering effects, and if these effects are associated with specific comorbidities or if there are race, age, or sex-specific effects.

Since the ultimate result of statins is the inhibition of L-mevalonate production through inhibiting hydroxymethylglutaryl-CoA reductase, this might be an underlying mechanism of antidepressant action. To further test the role of L-mevalonate in depression, future studies should also investigate amino-bisphosphonates which affect the same pathway. Amino-bisphosphonates inhibit L-mevalonate production, and have been used for osteoporosis, Paget’s disease, and cancer treatment [275,276], though their antidepressant or mood-altering effects have not been reported. The combination of amino-bisphosphonates with statins might lead to more L-mevalonate inhibition, which might increase the efficacy of the treatment.

### 5.4. Gut Microbiota

The symbiotic intestinal microbiota has been associated with several important processes in the body, including immunity and metabolism. More recently, an association between gut microbiota and depression has been uncovered, with significant gut microbiota variation between MDD and healthy patients [277]. Pre-clinical and clinical trials summarizing these findings will be reported in this section.

Disruption of microbiota is associated with increased depressive-like behaviors in mice. Fecal microbiota transplantation from chronic social defeat stress (CSDS)-susceptible mice increased depressive and anhedonia-like behaviors in recipient mice, along with an increase in IL-6 levels [278]. *Lactobacillus intestinalis* and *Lactobacillus reuteri* were suggested to be responsible for these depressive-like behaviors [278]. When similar microbiota transplantation was used in resilient Ephx2 knock-out mice, increased depressive-like behaviors, anhedonia, and IL-6 levels were reported [279]. *Faecalibaculum rodentium* was reported to cause the increase in stress susceptibility [279]. Mice subjected to antibiotic-induced microbiome depletion were resilient to CSDS-induced anhedonia. This antibiotic cocktail treatment increased proteobacteria with significant alterations in microbiota [280].

Prebiotic or probiotic treatment could be important to consider for the treatment of depression. Mice treated with the two prebiotics fructo-oligosaccharides (FOS) and galacto-oligosaccharides (GOS), either as mono or combination therapy, showed decreased depressive-like behaviors and anxiety-like behaviors [281]. Additionally, corticosterone release was decreased in GOS and FOS+GOS treated mice, with downregulation of proinflammatory cytokine levels reported in the latter group [281].

*Bifidobacterium* was detected in the gut of mice resilient to CSDS [282] and treatment with *Bifidobacterium* improved social interaction, normalized IL1-B levels in the PFC, and normalized the changes in gut microbiota composition [283]. Lower corticosterone levels were reported in the serum of mice treated with *Bifidobacterium breve* and increased butyrate levels were found in the cecum of mice treated with *Bifidobacterium longum* subsp. *infantis*. Both treatments enhanced chronic-stress-induced microbial dysbiosis [284].

In clinical studies, probiotic treatment significantly improved the mood of patients diagnosed with mild to moderate depression [285]. Patients with irritable bowel syndrome treated with the probiotic *Bifidobacterium longum* reported a significant reduction in depression score compared to the placebo group [286]. This score was also decreased in MDD patients treated with probiotic supplements, with downregulated serum hs-CRP, and increased plasma total glutathione in treated patients [287]. *Bifidobacterium longum 1714* administration significantly reduced stress levels, attenuated anxiety, and improved memory in healthy volunteers exposed to psychological and physiological stressors [288].

In additional studies, however, treatment with either probiotics or prebiotics did not significantly improve depressive symptoms or scores [289,290,291,292,293]. Either differences in responsiveness is due to the parameters of the study, or like other antidepressants, there are individuals who respond to some treatments, but not to others. This conflicting reporting stresses (1) the importance of further elucidating the etiology of depression and the rationale for individuals who are resilient and (2) the urgent need for additional randomized, double-blind, placebo-controlled trials with larger sample sizes to confirm or refute the antidepressant potential of pre/probiotics.

## 6. Potential Antidepressants from Natural Products

A remarkable number of plant extracts and natural products are reported to have antidepressant potential. Notably, few clinical trials have been conducted on natural products for most diseases, including psychiatric diseases. The lack of rigorous studies remains a major limitation in determining the efficacy of these potential therapeutic agents. Natural products that are reported to have preclinical or clinical antidepressant effects are discussed below and are summarized in Supplementary Tables S4 and S5.

### 6.1. Plants

Natural products are used by 75% of the world for routine health care, cancer treatment, multiple sclerosis, and infectious diseases. Many of these products including saponins, triterpenoids, and flavonoids have antidepressant potential through the inhibition of serotonin, norepinephrine, and dopamine transporters [294,295]. Others inhibit monoamine oxidase [296] and increase hippocampal dopamine levels [297,298]. Traditional Chinese medicinal plants that reduce depressive symptoms have a similar mechanism to antidepressant prescription drugs. Here, we will highlight the most recent preclinical in vivo studies demonstrating the antidepressant potential of natural products. For a comprehensive review of medicinal plants used for depression treatment, we recommend Moragrega et al. [299].

Plants used in traditional Chinese medicine were shown to target the 5-HT1a receptor [300] and transporter [301], and enhance synaptic plasticity through AMPAR and mTOR signaling [302]. Two flavonoids extracted from *Tilia americana*, a plant grown in North America that has been used to treat sleep disorders [303], showed significant antidepressant effects through the 5-HT1A and 5-HT2A receptors [304]. *Panax ginseng*, *Polygala tenuifolia,* Shen Yuan, and *Fructus aurantii,* are used in traditional Chinese medicine and were shown to activate BDNF and rescue rodents from depressive-like behaviors [305,306,307,308]. The herbal formula traditionally used in Thailand, Kleeb Bua Daeng, affected BDNF expression and proinflammatory factors in the PFC and the hippocampus [309]. Silexan, an essential oil extracted from lavender, delivered an antidepressant effect by increasing synaptogenesis and phosphorylation of the transcription factor cAMP response element-binding (CREB) [310].

The following plants and extracts are reported to have both anti-inflammatory and anti-depressant properties. *Schisandra chinensis* [311], helicid [312], panaxynol [313], and safflower extract [314] regulated various factors in the NF-κB and toll-like receptor 4–nucleotide-binding and oligomerization domain-like receptor 3 (NLRP3) signaling pathways. The honey fungus *Armillaria mellea* [315] reduced TNF-α and IL-1β [316]. *Radix polygalae* increased autophagy and reduced neuroinflammation [317]. *Schisandra chinensis* [311], *Cistanche tubulosa* [318], and *Sophora alopecuroides* [319] promoted diverse intestinal microbiome.

The role of PPAR in the antidepressant potential of natural products has been reported in carnosic acid, oridonin, and icariin. Carnosic acid [320] and oridonin [321] rescued mice from depressive-like behaviors by increasing PPAR-γ expression and adiponectin. Icariin, used in traditional Chinese medicine, attenuated M1 microglia in stressed mice by activating PPAR-γ. This resulted in reduced pro-inflammatory proteins, Aβ plaques, and M1 microglia in the hippocampus and the PFC of stressed mice [322].

Numerous plant extracts showed antidepressant-like effects in behavioral tests conducted on mice, but more research is needed to further study the specific mechanisms of action. These include: *Woodfordia fruticose* leaf extract [323], myrsinoic acid B from *Myrsine coriacea* [324], parsley [325], *Bacopa monnieri (Linn.) Wettst* [326], *Salvia officinalis,* and *Lippi triphylla* [327].

Plant extracts can target multiple receptors, distinct biological processes, or have widespread molecular and behavioral effects [299]. Several plant extracts have behavioral effects similar to current antidepressant drugs [328]. However, these antidepressant effects are often reported without a proposed underlying mechanism. Therefore, further studies on efficacious plant extracts should be conducted to determine mechanisms underlying antidepressant effects. For some plant extracts, there are proposed mechanisms of action, such as helicid and *Sophora alopecuroides*. Antidepressant effects could be produced by a reduction in M1 microglia or reduction of inflammatory factors [312,329]. These products should be further explored in rigorous clinical studies to determine their efficacy in humans. 

### 6.2. N-3 Polyunsaturated Fatty Acids

The cell membrane is composed of polyunsaturated fatty acids (PUFAs), which have a crucial role in physiological, neuronal, and metabolic function [330]. PUFAs are classified as N-3 and N-6 fatty acids and are thought to promote neurogenesis, anti-inflammatory effects, and to modulate neurotransmission [331]. Important PUFAs including eicosapentaenoic acid (EPA), arachidonic acid (AA), and docosahexaenoic acid (DHA) are needed to biosynthesize endocannabinoids, endogenous ligands of the cannabinoid receptors that play a crucial role in synaptic plasticity and CNS development [332]. Imbalance in these acids often leads to neuroinflammation in important brain regions [333]. Additionally, dysregulation of DHA/EPA or AA levels negatively impacts serotonin levels and limbic system regulation by the frontal cortex [334]. Contradictory evidence currently exists, and more studies are necessary to understand the complexities and potential of PUFAs as antidepressant agents. Here, we provide current clinical studies that primarily demonstrate the antidepressant effects of PUFAs.

Treatment with N-3 PUFAs has improved depressive symptoms in elderly patients, patients with acute myocardial infarction, and patients diagnosed with heart-failure depression [335,336,337]. N-3 PUFAs antidepressant effect was delivered in a dose-dependent manner [338]. Additionally, combinatorial therapy of N-3 PUFAs and ascorbic acid [339] or sertraline [340] improved symptoms of depression. EPA and DHA reduced depressive symptoms with a superior overall clinical benefit of EPA compared to DHA [341,342,343].

There are numerous clinical reports of no significant antidepressant effect from PUFAs [344,345,346,347,348,349]. Interestingly, a majority of these studies were conducted in children, adolescents, or elderly populations. Bai et al. reported mixed findings on the antidepressant potential of N-3 PUFAs in treating MDD elderly patients [350]. In patients with cardiovascular diseases comorbid MDD, no significant overall effect was reported on depressive symptoms with N-3 PUFAs treatment compared to placebo. However, this treatment reduced core depression symptoms in severe MDD patients [351], suggesting that N-3 PUFAs treatment may be more effective in treatment-resistant populations.

There are currently an equal number of studies indicating the antidepressant effect of N-3 PUFAs as there are studies without an antidepressant effect. The difference in responsiveness to N-3 PUFAs suggests it may be important to consider specific demographics such as age and other health issues of the patient. Although DHA supplementation is a common practice and available over-the-counter, the clinical benefit and therapeutic dosage remain unclear and must be confirmed with controlled clinical trials. As such, prioritizing clinical trials should be considered for MDD patients, or for individuals who have risk factors for developing MDD.

### 6.3. Resolvins

Resolvins are molecules belonging to the family of specialized pro-resolving lipid mediators [352]. Enzymatically produced in organs and fluids, resolvins are derived from acids including DHA (RvD1 and RvD2) and EPA (RvE1, 2 and 3) [353]. RvDs and RvEs were recently shown to induce antidepressant effects in multiple pre-clinical studies discussed below.

Intracerebroventricular (ICV) injection, PFC infusion, or hippocampal infusion of RvD1, RvD2, or AT-RvD1 reduced depressive-like behaviors [354,355,356]. This antidepressant effect was reversed when two antagonists, formyl peptide receptor 2/lipoxin A_4_ receptor (FPR2/ALX) and GPR18, were administered, or with mammalian target of rapamycin complex 1 inhibition by rapamycin [355]. Cultured hippocampal neurons had reduced dendritic and axonal length when treated with FPR2/ALX [357]. RvD1 activated the AMPAR and PI3K pathways, while RvD2 activated the mitogen-activated protein kinase kinase (MEK)-ERK pathway [355].

Similar outcomes were reported with EPA-derived resolvins. LPS-induced depressive-like behaviors in mice were reduced after RvE1 and RvE2 ICV injections [358,359]. A ChemR23 (G protein-coupled receptor) agonist reversed depressive-like behaviors, suggesting that ChemR23 agonistic activity is important for RvE1 and RvE2 antidepressant potential [358].

Altogether, these studies demonstrate the antidepressant potential of resolvins. Because administration has primarily been through ventricle or direct brain injection, an important step will be in determining the dosage and efficacy of more convenient delivery methods. As mentioned above, resolvins are derived from DHA, further supporting an exploration into the benefits of DHA supplementation.

## 7. Conclusions

Here, we have summarized the most recently studied pharmaceutical approaches, including synthetic compounds and natural products to target receptors or biological processes. Current antidepressant treatment options are inadequate for patients, primarily due to adverse effects, delayed onset of antidepressant action, and inadequate efficacy.

One of the major issues underlying inadequate pharmacological treatment is due to an incomplete understanding of the etiology of depression. First-line antidepressants primarily target the serotonin or norepinephrine system, yet it is apparent that molecular mechanisms underlying depression extend beyond monoamines. Prioritizing research efforts on elucidating mechanisms underlying resilience or susceptibility to depression and identifying genetic or epigenetic risk factors is a critical first step to developing novel therapeutics.

MDD is a heterogenous disease; patients experience unique combinations of symptoms and therefore it is expected that patients would have variable responses and require personalized treatment. Increasing the available pharmaceutical agents will allow clinicians to overcome current obstacles by personalizing treatment based on the patient’s symptoms, risk factors, sex, age, response to prior treatment, and other important factors. As more molecular modulators are identified to have a role in the development of depression, it is important to consider combinatorial therapy addressing multiple dysregulated factors involved in the process.

There are numerous studies that indicate probiotics and natural products have antidepressant potential, yet confounding evidence in both preclinical and clinical studies still exists. One of the major limitations in determining efficacy is the severe lack of large-scale placebo controlled, double-blinded clinical studies. By conducting these studies, more evidence could be generated, which might strengthen the use of these natural products as potential approaches for MDD treatment. Combinatorial therapy can also be tested, which might lead to an enhanced treatment efficacy.

Many of the novel targets described in this review are in early preclinical studies phases of discovery. Prioritizing research on these novel mechanisms could progress to rigorous preclinical and clinical studies in the future, and ultimately lead to the identification of novel targets and improved treatment options.

## Supplementary Materials

The following supporting information can be downloaded at: https://www.mdpi.com/article/10.3390/life12020196/s1, Table S1: Receptor Targeted in Preclinical Studies; Table S2: Receptors Targeted in Clinical Studies; Table S3: Biological Processes Targeted in Preclinical Studies; Table S4: Biological Processes Targeted in Clinical Studies; Table S5: Preclinical Studies on Natural Products; Table S6: Clinical Studies on Natural Products.
