# Supplementary material for: Novel Pharmacological Approaches to the Treatment of Depression
Source: Life (Basel). 2022 Jan 28;12(2):196. doi: 10.3390/life12020196 (PMC8879976; doi:10.3390/life12020196)
Supplement: Supplementary file 1 [file life-12-00196-s001.zip › life-1501669-supplementary.pdf]

Target	Mechanism of Action	Compound	Subjects	Assessment	References
**Opioid Receptor**	KOR and DOR ligand	Enkephalins	ENK knockout mice; UCMS	EZM, FST, SPT	Melo et al., 2014
	DOR agonist	KNT-127	ICR mice	FST	Saitoh et al., 2011
		SNC80	NMRI mice; SIS	FST	Mirzaian et al., 2019
		Rubiscolin-6	C57BL/6J mice; CRS	TST	Mitsumoto et al., 2019
	MOR antagonist, partial KOR, DOR agonist	Nalmefene	Wistar Hannover rat; IFN-α treatment	EPM, FST, OET	Callaghan et al., 2018
	NOR antagonists	LY2940094	NOR knockout mice	FCT, FST	Witkin et al., 2016
		UFP-101	Wistar rats; UCMS	FST, OFT, SCT	Vitale et al., 2017
	MOR agonist	Tianeptine	C57BL/6J mice; Antidepressant treatment	NSF, OFT	Pekarskaya., et al 2021
			Wistar rats; Chronic SIS	FST, Marble burying, SPT	Perić et al., 2020
			Sprague Dawley rats; Prenatal stress	EPM, FST	Trojan et al., 2019
			Albino Swiss mice; Antidepressant treatment	FST, TST	Poleszak et al., 2020
**NMDAR**	NMDAR agonist	AGN-241751	C57BL/6J mice; UCMS	FUST, NSF, SST	Pothula et al., 2020
		DAPK1	Sprague Dawley rats; UCMS, DAPK1 knockdown	FST, SPT	Li et al., 2018
	NMDAR antagonist	MK-801	C57BL/6J mice; BCG vaccine	FST, SPT, TST	Rana et al., 2020
			C57BL/6 mice, CD1 mice; CSDS	FST, SPT, TST	Yang et al., 2016
		Ro 25-6981	C57BL/6J mice	TST	Yellepeddi et al., 2018
		Rislenemdaz	C57BL/6J mice; UCMS	FST, OPT, SPT	Lei et al., 2020
		Traxoprodil	Albino Swiss mice; Antidepressant treatment	FST	Poleszak et al., 2016
			Albino Swiss mice; Antidepressant treatment	FST	Stasiuk et al., 2017
		Lanicemine	Swiss mice; UCMS	NSF, TST	Neis et al., 2020
		Nitrous oxide	CD-1 mice	FST, TST	Liu et al., 2020
		Ifenprodil	Sprague-Dawley rat; UCMS	FST, SPT	Yao et al., 2020
**PPAR**	PPAR agonist	Pioglitazone	C57BL/6N mice; high-fat diet	FST, TST, SPT	Lam et al., 2021
			Rats; LPS	EMP, FST, OPT	Beheshti et al., 2019
		Rosiglitazone	Wistar rats; UCMS	Coat state, EPM, NOR, OFT, SST	Keledjian et al., 2020
**GPR39**	GPR agonist	TC-G 1008	CD-1 mice	FST, OFT	Starowicz et al., 2019
		Ghrelin	Sprague Dawley rats; Myocardial infarction induction	EPM, OFT, SPT	Sun et al., 2021

**mGluR**	mGluR 2/3 antagonist	LY3020371	Sprague-Dawley rats	FST	Witkin et al., 2017
		LY341495	C57BL/6J mice; UCMS	FST, OFT, Rotarod test, SPT, SST, TST,	Pałucha-Poniewier et al., 2021
	mGluR2/3 NAM	VU6001966 and VU0650786	C57BL/6J mice	FST, SPT	Joffe et al., 2020
**Galanin Receptor**	Ligand	GAL 1-15	Sprague Dawley rats; Equitesin injection	FST, LDT, OFT, TST	Millon et al., 2015
			Sprague Dawley rats; 8-OH-DPAT Injection	FST	Millon et al., 2016
	GAL2 agonist	AR-M1896	Wistar rats; M871 Treatment	FST	Souza et al., 2018
	Active galanin analog	J18	C57BL/6J mice; Peptides and imipramine injection	TST	Saar et al., 2018

Table S1. Receptors Targeted in Preclinical Studies
Abbreviations: BCG, Bacillus Calmette-Guerin; CRS, chronic restraint stress; CSDS, chronic social defeat stress; EPM, elevated plus maze; EZM, zero maze test; FCT, fear conditioning test; FST, forced swim test; FUST, female urine sniffing test; LDT, light dark test; LPS, lipopolysaccharide; MWM, Morris water maze task; NOR, novel object recognition; NSF, novelty suppressed feeding; OET, object exploration task; OFT, open field test; SPT, sucrose preference test; SCT, sucrose consumption Test; SIS, social isolation stress; SST, sucrose splash test; TST, tail suspension test; UCMS, unpredictable chronic mild stress.

**Target**	**Mechanism of Action**	**Compound**	**Subjects**	**Assessment**	**References**
**Opioid Receptor**	Partial MOR agonist, DOR, KOR antagonist	Buprenorphine	TRD patients	MADRS	Zajecka et al., 2020
			TRD patients over 50 years old	HVLT-R, MADRS	Karp et al., 2014
**NMDAR**	NMDAR antagonist	Nitrous oxide	TRD patients	HDRS	Nagale et al., 2015
		Memantine	Depressive patients over 60 years old	HDRS, MADRS	Lavretsky et al., 2021
		Amantadine	BDV-1 infected patients with MDD or bipolar disorder	HDRS	Dietrich et al., 2020
		Riluzole	MDD patients	HDRS	Salardini et al., 2021
**GPR39**	GPR agonist	Basimglurant	MDD patients	MADRS	Quiroz et al., 2016

Table S2. Receptors Targeted in Clinical Studies
Abbreviations: HDRS, Hamilton depression rating scale; HVLT-R, Hopkins verbal learning test-revised; MADRS, Montgomery-Åsberg depression rating scale.

Target	Mechanism of Action	Compound	Subjects	Assessment	References
**Inflammation**	COX-2 inhibitor	Ibuprofen	Wistar rats; CRS	FST, PAM	Nozari et al., 2020
			Sprague Dawley rats; CRS	FST	Seo et al., 2019
		Nimesulide	Sprague-Dawley rats UCMS	FST, OFT	Luo et al., 2020
		Lumiracoxib	ICR mice; Corticosterone treatment	EPM, LDT	Morgan et al., 2019
	Ketamine metabolite	(2R,6R)-HNK	CD-1 Mice, C57BL/6J Mice, GRM2/3 knockout mice; CSDS	FST	Zanos et al., 2019
	Akt/NF-κB inhibitor	Modafinil	C57BL/6 mice	FST, MWM	Han et al., 2018
	TNF-α antagonist	Etanercept	Long Evans rat	FST	Brymer et al., 2018
	Antioxidant	NAC	Wistar rats; Clomipramine treatment	NSF, SPT	Chakraborty et al., 2020
			Wistar rats; UCMS	FST, SPT	Fan et al., 2020
**HPA axis**	GR Regulator	FKBP5	MDD patients	Blood analysis	Ising et al., 2019
			FKBP5 knockout	SPT	Kwon et al., 2019
		Ahi1gene	C57BL/6J and DBA/2 inbred mice	AFL, MWM, NOR	Lotan et al., 2016
			C57BL/6J mice; Antidepressant treatment	FST, TST	Xu et al., 2010
			ICR mice	FST, TST	Wang et al., 2021
	MR agonist	Fludrocortisone	Depressive patients	Neuropsychol ogical assessment	Otte et al., 2015
**Cholesterol metabolism**	HMG-CoA reductase inhibitors	Simvastatin	C57BL/6J mice; LPS, UCMS	FST, NSF, SPT,	Yu et al., 2019
			C57BL/6J mice; High-fat diet	OFT, TST	Wu et al., 2019
			Sprague Dawley rats; Carprofen treatment	FST, SST	Menze et al., 2021
		Atorvastatin	Swiss albino mice; LPS	FST, TST	Taniguti et al., 2019
			C57BL/6 mice; Streptozotocin treatment	NSF, SPT, TST	Hai-Na et al., 2020
			C57BL/6 mice; Streptozotocin treatment	EPM, OFT, Rotarod test, TST	Yan el al., 2021
		Atorvastatin & simvastatin	Swiss albino mice; Agmatine treatment	FST	Rahangdale et al., 2021
		Lovastatin	C57BL/6J mice; Streptozotocin treatment	NSF, SPT, TST	Tang et al., 2020
**Gut microbiota**	Prebiotics	FOS and GOS	C57BL/6J mice	TST	Burokas et al., 2017
	Probiotics	*Bifidobacterium*	C57BL/6J mice; CSDS	SIT, SPT	Yang et al., 2017
		Bifidobacterium Longum Subsp. Infantis E41 And	C57BL/6J mice; UCMS	FST, Step-down tests, SPT	Tian et al., 2019

		Bifidobacterium Breve M2CF22M7			
		Microbiome	C57BL/6 mice; Antibiotic treatment, CSDS, microbiome depletion	SPT	Wang et al., 2020

Table S3. Biological Processes Targeted in Preclinical Studies
Abbreviations: Ahi1, Abelson helper integration site 1; AFL, associative fear learning; FOS, fructo-oligosaccharides; GOS, galacto-oligosaccharides; NAC, N-Acetyl Cysteine; PAM, Passive avoidance memory test.

Target	Mechanism of Action	Compound	Subjects	Assessment	References
**Inflammation**	COX-2 inhibitor	Celecoxib	MDD patients	HDRS	Majd et al., 2015
				MADRS	Fourrier et al., 2018
	COX-1/2 inhibitor	Naproxen	Osteoarthritis patients	PHQ-9	Iyengar et al., 2013
		Diclofenac	Chronic pain patients	HADS, HDRS	Jafarinia et al., 2016
		Aspirin	MDD patients	MADRS	Savitz et al., 2018
				CES-D-10	Berk et al., 2020
	IL-12/23 inhibitor	Ustekinumab	Psoriasis patients	BDI, HDRS	Kim et al., 2018
	TNF antagonist	Adalimumab	Hidradenitis suppurativa patients	PHQ	Scheinfeld et al., 2016
		Guselkumab	Psoriasis patients	HADS	Reich et al., 2020
	TNF-α antagonist	Infliximab	Depressive and bipolar patients	NEVs biomarker	Mansur et al., 2020
	Antibiotic	Minocycline	MDD patients	HDRS	Nettis et al., 2021
			Bipolar depression patients	MADRS	Murrough et al., 2018
			MDD patients	HDRS, MADRS	Zazula et al., 2020
	IL-6 antagonist	TCZ	Rheumatoid arthritis patients	HDRS	Tiosano et al., 2020
**HPA axis**	GR Regulator	Mifepristone	Alcohol dependent patients with depressive symptoms	BDI	Donoghue et al., 2020
			Psychotic depression patients	BPRS, HDRS	Block et al., 2018
		Prednisone & Dexamethasone	MDD patients	Cortisol assessment	Barroca et al., 2021
	V1B antagonist	ABT-436	Depressive patients	HDRS-17, MADRS	Katz et al., 2017
		TS-121	TRD patients	MADRS	Kamiya et al., 2020
	MR antagonist	Spironolactone	Healthy volunteers	MRI	Vogel et al., 2017
**Gut microbiota**	Probiotics	Bifidobacterium Longum 1714	Healthy volunteers	PSS, EEG, PAL	Allen et al., 2016
		Bifidobacterium Longum	Patients with irritable bowel syndrome	HDRS	Pinto-Sanchez et al., 2017
		Lactobacillus Acidophilus,	MDD patients	BDI	Akkasheh et al., 2016

		Lactobacillus Casei and Bifidobacterium Bifidum			

Table S4. Biological Processes Targeted in Clinical Studies
Abbreviations: BDI, Beck depression inventory; BPRS, brief psychiatric rating scale; CES-D-10, center for epidemiologic studies depression 10-item scale; HADS, hospital anxiety and depression scale; NEVs, neuronal origin of blood extracellular vesicles; PAL, paired associate learning task; PHQ-9, patient health questionnaire; PSS, Cohen perceived stress scale; TCZ, tocilizumab; V1B, vasopressin type 1B receptor

Source	Natural Product	Subjects	Assessment	References
Plants	Silexan oil (Lavender)	Sprague Dawley rats	FST	Friedland et al., 2021
	Fructus aurantii	ICR mice; LPS and LH	LH, NSF, FST, SPT, TST	Wu et al., 2021
	Armillaria mellea	Sprague Dawley rats; UCMS	FST, SPT	Lin et al., 2021
	Radix polygalae	ICR mice and Wistar rats; CRS	FST, NSF, OFT	Zhou et al., 2021
	Tilia americana	ICR mice	FST	Martínez-Hernández et al., 2021
	Panax ginseng and *Polygala tenuifolia*	Sprague Dawley rats; UCMS	FST, NSF, SPT, TST	Jiang et al., 2021
	Uncaria rhynchophylla	C57BL/6 mice; UCMS	EPM, FST, SPT, TST	Qiao et al., 2021
	Gardeniae fructus	BALB/c mice; SRS	FST, SPT, TST	Xia et al., 2021
	Safflower	ICR mice; UCMS	FST, SPT, TST	Chen et al., 2021
	Schisandra chinensis	C57BL/6 mice; LPS	FST, TST	Yan et al., 2021
	Sophora alopecuroides	ICR mice; UCMS	FST, OFT, SPT	Zhang et al., 2021
	Shen Yuan	C57BL/6 mice; CSDS	FST, SIT, SPT, TST	Jiang et al., 2021
		Sprague Dawley rats; UCMS	FST, SPT, TST	Jiang et al., 2021
	Nardostachys jatamansi	ICR mice	OFT, TST	Li et al., 2021
	Carnosic acid	ICR mice and adipo knockout mice; UCMS	FST, NFT, OFT, SPT	Wang et al 2021
	Cistanche tubulosa	Sprague Dawley rats; UCMS	FST, SPT	Fan et al., 2021
	Parsley polyphenols	Swiss albino mice	FST, LDT, OFT	Es-Safi et al., 2021
	Helicid	Sprague Dawley rats; UCMS	FST, OFT, SPT	Zhang et al., 2021
	Kleeb Bua Daeng	ICR mice; UCMS	FST, SPT, TST	Maneenet et al., 2021
	Panaxynol	ICR mice; LPS	EPM, FST, OFT, TST	Zhao et al., 2021
	Salvia officinalis and *Lippia triphylla*	Swiss albino mice	FST, TST	Maliki et al., 2021
	Woodfordia fruticosa	Swiss albino mice	FST, TST	Tayab et al., 2021
	Oridonin	CD-1 mice	FST, TST	Liu and Du, 2020
	Myrsinoic acid B from *Myrsine coriacea*	Wistar rats	FST, OFT	Zimath et al., 2021
	Icariin	APP/PS1 mice; restraint/isolation stress	MWM, SPT	Wang et al. 2019
Omega-3 Fatty Acids	RvD1, AT-RvD1 and RvD2	Swiss mice; fibromyalgia induction	Hot-plate, Mechanical allodynia	Klein et al., 2014
	RvD1 and RvD2	BALB/c mice; LPS	TST	Deyama et al., 2017

		BALB/c mice; UCMS	FST, TST	Ishikawa et al., 2017
	RvE1 and RvE2	BALB/c mice; LPS	FST, LAT, TST	Deyama et al., 2018
	RvE3	BALB/c mice; LPS	TST	Deyama et al., 2018
	N-3 PUFAs	Patients with AMI	STAI	Haberka et al., 2013

Table S5. Preclinical Studies on Natural Products

Abbreviations AMI, acute myocardial infarction; CHF, chronic heart failure; SRS, spatial restraint stress; STAI, State-Trait Anxiety Inventory

Source	Natural Product	Subjects	Assessment	References
**Omega-3 Fatty Acids**	N-3 PUFAs	Depressive patients with CHF	HDRS	Jiang et al., 2018
	N-3 PUFAs with setraline	MDD patients	MADRS	Jahangard et al., 2018
	N-3 PUFAs with ascorbic acid	Depressive rotational shift workers	BDI	Khajehnasiri et al., 2016

Table S6. Clinical Studies on Omega-3 Fatty Acids
